# Downregulation of renal tubular Wnt/*β*-catenin signaling by Dickkopf-3 induces tubular cell death in proteinuric nephropathy

**DOI:** 10.1038/cddis.2016.62

**Published:** 2016-03-24

**Authors:** D W L Wong, W H Yiu, H J Wu, R X Li, Y Liu, K W Chan, J C K Leung, L Y Y Chan, K N Lai, S C W Tang

**Affiliations:** 1 Division of Nephrology, Department of Medicine, The University of Hong Kong, Queen Mary Hospital, Hong Kong; 2 The University of Hong Kong Shenzhen Institute of Research and Innovation, China

## Abstract

Studies on the role of Wnt/*β*-catenin signaling in different forms of kidney disease have yielded discrepant results. Here, we report the biphasic change of renal *β*-catenin expression in mice with overload proteinuria in which *β*-catenin was upregulated at the early stage (4 weeks after disease induction) but abrogated at the late phase (8 weeks). Acute albuminuria was observed at 1 week after bovine serum albumin injection, followed by partial remission at 4 weeks that coincided with overexpression of renal tubular *β*-catenin. Interestingly, a rebound in albuminuria at 8 weeks was accompanied by downregulated tubular *β*-catenin expression and heightened tubular apoptosis. In addition, there was an inverse relationship between Dickkopf-3 (Dkk-3) and renal tubular *β*-catenin expression at these time points. *In vitro,* a similar trend in *β*-catenin expression was observed in human kidney-2 (HK-2) cells with acute (upregulation) and prolonged (downregulation) exposure to albumin. Induction of a proapoptotic phenotype by albumin was significantly enhanced by silencing *β*-catenin in HK-2 cells. Finally, Dkk-3 expression and secretion was increased after prolonged exposure to albumin, leading to the suppression of intracellular *β*-catenin signaling pathway. The effect of Dkk-3 on *β*-catenin signaling was confirmed by incubation with exogenous Dkk-3 in HK-2 cells. Taken together, these data suggest that downregulation of tubular *β*-catenin signaling induced by Dkk-3 has a detrimental role in chronic proteinuria, partially through the increase in apoptosis.

Chronic kidney disease (CKD) is a global public health problem affecting approximately 10% of the world population.^1^ Despite its diverse etiologies, progressive CKD is characterized histologically by a common pathway of renal tubulointerstitial damage.^2^ One established link between the different forms of glomerular disease and lesions in the interstitial compartment is proteinuria.

Notably, albumin is the most abundant protein in the glomerular filtrate, which is reabsorbed by the proximal tubule via receptor-mediated endocytosis.^3, 4^ This process activates a range of intracellular signaling pathways in renal cells^5, 6, 7^ and triggers the tubular epithelial cells to enter a proinflammatory state^8^ that heralds a profibrotic microenvironment with accumulation of extracellular matrix.^9^ Abnormal protein trafficking is also a causative factor of renal tubular cell death.^10, 11, 12^ An increasing number of experimental investigations and clinical observations have proved renal tubular cell apoptosis in the pathogenesis of renal tubular injury.^13, 14, 15^ Emerging evidence indicates that some underlying tubulotoxic mechanisms of tubular cell death are stimulated by albumin overload.^16^

The canonical Wnt/*β*-catenin signaling pathway participates in multiple physiological events associated with organ repair and developmental processes.^17, 18^ Emerging evidence suggests that the Wnt/*β*-catenin signaling pathway may be involved in different organ-specific diseases.^19, 20^ In the canonical Wnt/*β*-catenin signaling pathway, Wnt molecules in the extracellular matrix transmit the intracellular signal through interacting with Frizzled receptors and co-receptor LDL receptor-related protein (Lrp) 5/6.^21, 22^ This interaction elicits an intracellular signaling cascade resulting in an accumulation of non-phosphorylated *β*-catenin. The stabilized non-phosphorylated *β*-catenin translocates into the cell nucleus and works together with T-cell factor (TCF)/lymphoid enhancer-binding factor (LEF) transcription factors to trigger the transcription of Wnt target genes. Hence, the persistence of intracellular *β*-catenin accumulation increases the activity of Wnt/*β*-catenin signaling pathway. Recently, the Dickkopf family has been recognized as an important regulator of this signaling pathway, but little is known for its involvement in proteinuric nephropathy.^23^

In the kidney, canonical Wnt/*β*-catenin signaling pathway has been implicated in cystic kidney disease,^24, 25^ acute kidney injury (AKI) and diabetic nephropathy (DN).^26^ Recent evidence have indicated that aberrant Wnt/*β*-catenin activities in renal cells contributed to epithelial–mesenchymal transition (EMT),^27^ fibrosis^28, 29^ and apoptosis during nephropathy.^30^ However, data on the role of Wnt/*β*-catenin signaling in proteinuric nephropathy remains scarce. In this study, we presented *in vitro* and *in vivo* evidence that protein overload induced apoptosis in proximal tubular epithelial cells (PTECs) via downregulation of Wnt/*β*-catenin signaling pathway. In addition, the upregulation of Dickkopf-3 (Dkk-3) may act as a novel autocrine mechanism that suppresses Wnt/*β*-catenin signaling upon protein overload in PTECs.

## Results

### Downregulation of *β*-catenin expression in protein overloaded HK-2 cells

**Figure 1:**
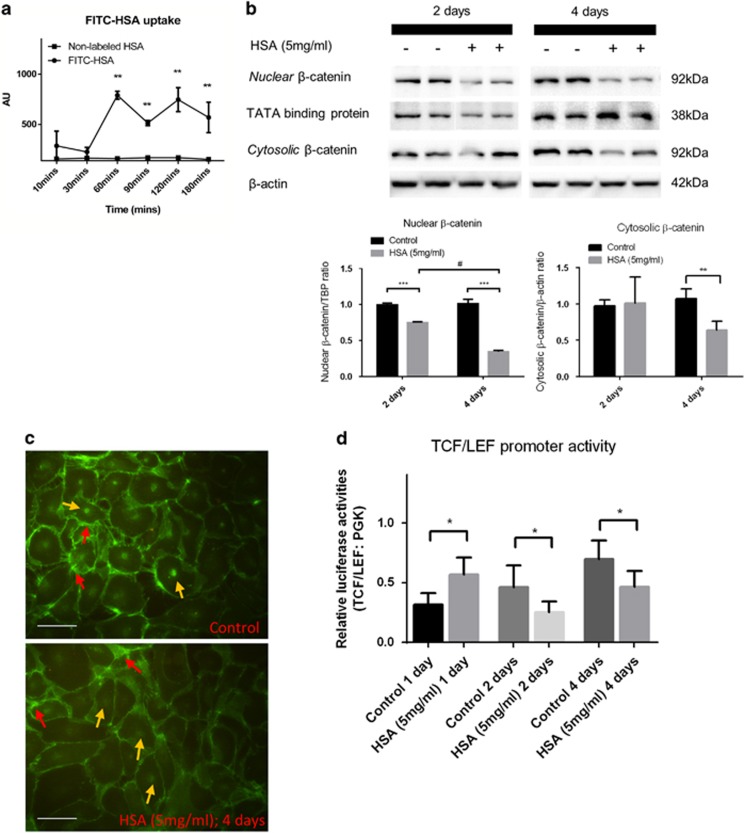
*β*-Catenin expression is abrogated in protein overloaded HK-2 cells. (**a**) Albumin uptake measurement (*n*=3) in lysed HK-2 cells treated with FITC-human albumin/non-labeled HSA. (**b**) HK-2 cells were treated with 5 mg/ml HSA for 2 (*n*=6) and 4 (*n*=6) days. Immunoblotting assays of active *β*-catenin expression in the nuclear fraction and cytosolic fraction of the cell lysate. (**c**) Immunofluorescent staining of *β*-catenin in HSA-treated HK-2 cells for 4 days. Red arrows show the membrane-bound *β*-catenin and yellow arrows indicate nuclear *β*-catenin. (**d**) Luciferase assay of TCF/LEF reporter activities in HSA-treated or control HK-2 cells. Bar scale=25 *μ*m. Graphs were expressed in mean± S.D. *P<0.05, **P<0.001, ***P<0.0001; ^#^*P*<0.01

We first demonstrated albumin uptake in human kidney-2 (HK-2) cells after 60-min incubation with fluorescein (FITC)-labeled human serum albumin (HSA; Figure 1a). In HSA-treated HK-2 cells, activation of Wnt/*β*-catenin signaling was evident by the translocation of stabilized *β*-catenin into cell nuclei. After 2 days of treatment, HSA-treated cells showed significant downregulation of active *β*-catenin expression in the cell nucleus. By day 4, the reduction of both nuclear and cytosolic *β*-catenin expression was further decreased to approximately 50% compared with untreated HK-2 cells (Figure 1b). Immunofluorescence staining confirmed the localization of *β*-catenin at both cell membrane and nuclei of control HK-2 cells, whereas the signal intensity abated in protein overloaded cells (Figure 1c). Luciferase reporter assay demonstrated downregulation of TCF/LEF promoter activities in HK-2 cells after 2 and 4 days of HSA treatment, following a transient upregulation of promoter activity at day 1 (Figure 1d). These data together demonstrated the abrogation of Wnt/*β*-catenin signaling pathway in HK-2 cells upon prolonged stress from protein overload.

### Upregulation of Dkk-3 expression in protein overloaded HK-2 cells suppressed Wnt/*β*-catenin signaling

**Figure 2:**
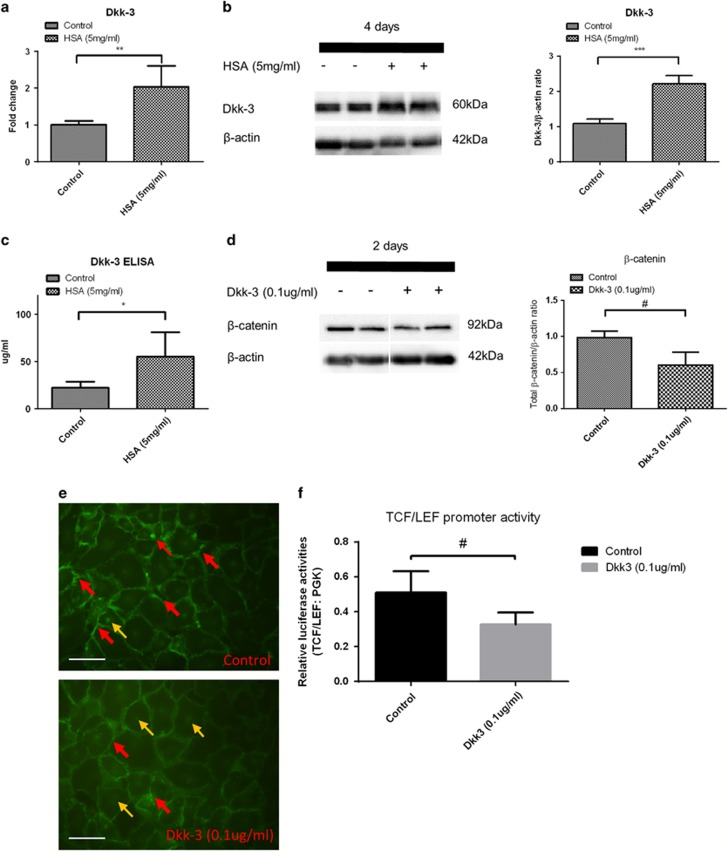
Dkk-3 expression is elevated in protein overloaded HK-2 cells that suppresses intracellular *β*-catenin expression. HK-2 cells were treated with 5 mg/ml HSA for 2 and 4 days. (**a**) Dkk-3 gene expression in the HSA-treated HK-2 cells for 2 days by quantitative real-time PCR (*n*=8). (**b**) Dkk-3 levels in HSA-treated HK-2 cell lysate for 4 days by immunoblotting (*n*=8). (**c**) Dkk-3 protein levels in HK-2 cell culture supernatants at 4 days after HSA treatment (*n*=5). (**d**) Change in total *β*-catenin levels in HK-2 cells by immunoblotting (*n*=8) after exposure to 0.1 *μ*g/ml human recombinant Dkk-3 protein for 2 days. (**e**) Immunofluorescent staining of *β*-catenin in HK-2 cells treated with Dkk-3. Red arrows show the membrane-bound *β*-catenin and yellow arrows indicate nuclear *β*-catenin. (**f**) Luciferase assay of TCF/LEF reporter activities in Dkk-3 treated or control HK-2 cells. Bar scale=25 *μ*m. Graphs were expressed in mean±S.D. *P<0.05, **P<0.001, ***P<0.0001, ^#^*P*<0.01

Dickkopf proteins, consisting of isotypes Dkk-1 to Dkk-4, provide feedback control of Wnt/*β*-catenin signaling. We postulated that these molecules would be upregulated by protein overload in HK-2 cells. As shown in Figures 2a and b, expression of Dkk-3 was upregulated in at both gene and protein level in HSA-treated HK-2 cells. Importantly, extracellular Dkk-3 has been reported to suppress Wnt/*β*-catenin signaling.^31^ We also showed a twofold increase in secreted Dkk-3 protein levels in culture medium by ELISA assay (Figure 2c).

Treatment of HK-2 cells with human recombinant Dkk-3 protein reduced total *β*-catenin protein expression (Figures 2d and e). Exogenous Dkk-3 also downregulated the TCF/LEF promotor activity in HK-2 cells (Figure 2f). Notably, these phenotypes recapitulated those of the HSA treatment, suggesting that Dkk-3 overexpression may mediate the effect of protein overload in HK-2 cells via an autocrine manner.

### Protein overload induced apoptosis in HK-2 cells via downregulation of Wnt/*β*-catenin signaling

**Figure 3:**
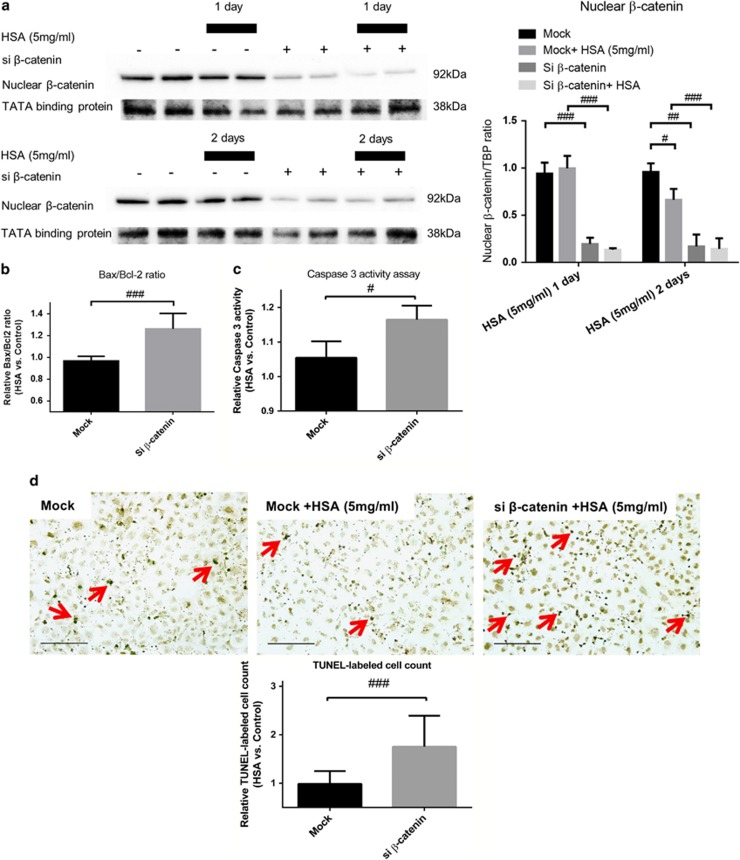
Loss of *β*-catenin/HSA stimulation promotes apoptosis in HK-2 cells. HK-2 cells were transfected with siRNA against *β*-catenin (si *β*-catenin) or mock siRNA (40 nM) for 2 and 3 days with or without addition of HSA (5 mg/ml) for 1 and 2 days. (**a**) Protein levels of nuclear *β*-catenin in transfected HK-2 cells treated with HSA for 1 day (*n*=3) and 2 days (*n*=6). (**b**) Bax/Bcl-2 gene expression in mock/si *β*-catenin transfected HK-2 cells under HSA stimulation for 2 days (*n*=6). (**c**) Caspase-3 activity in the corresponding groups of HK-2 cells after 2-day HSA treatment (*n*=3). (**d**) TUNEL-positive cells after 1-day HSA stimulation in HK-2 cells with or without si *β*-catenin transfection (*n*=6). Red arrows indicate apoptotic nuclei. Bar scale=25 *μ*m. Graphs were expressed in mean± S.D. ^#^*P*<0.05, ^##^*P*<0.001, ^###^*P*<0.0001

The role of Wnt/*β*-catenin signaling pathway in protein overloaded HK-2 cells was investigated using silencing RNA (siRNA)-mediated gene silencing of *β*-catenin. The expression of *β*-catenin was decreased gradually after 2 days in HSA-treated HK-2 cells, whereas the suppression of *β*-catenin was markedly enhanced and sustained in cells transfected with siRNA (Figure 3a).

Protein overload induced apoptosis in HK-2 cells after HSA treatment as indicated by an increase in gene expression ratio of Bax/Bcl-2, and the Bax/Bcl-2 ratio was further increased by silencing *β*-catenin (Figure 3b). To confirm the induction of apoptosis, we showed that the caspase-3 activities in these experimental groups had a similar trend to that of terminal deoxynucleotidyl transferase dUTP nick-end labeling (TUNEL) assay (Figure 3c). In line with these observations, the number of TUNEL-positive cells was significantly higher in HSA-treated HK-2 cells compared with untreated cells. The number of apoptotic cells was further increased after *β*-catenin gene silencing (Figure 3d). These results indicated that suppression of Wnt/*β*-catenin signaling exaggerated apoptosis in protein overloaded HK-2 cells.

### Kidney injury in the mouse model of overload proteinuria

**Figure 4:**
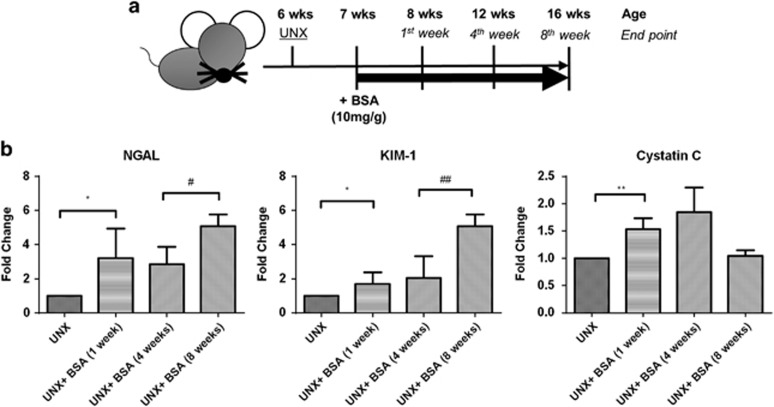
Induction of protein overload in C57/BL6 mice and longitudinal changes in AKI markers. (**a**) Schema of protein overload model. (**b**) Renal cortical expression of AKI markers (*n*=6) at the respective time points. Graphs were expressed in mean±S.D. *P<0.05, **P<0.001; ^#^*P*<0.05, ^##^*P*<0.001

Murine protein overload model was established in 6-week-old C57/BL6 mice that underwent uninephrectomy, and followed by a high dose of bovine serum albumin (BSA; 10 mg/g body weight) or saline injection for 8 weeks (Figure 4a). Uninephrectomized mice receiving BSA exhibited significantly higher urine albumin-to-creatinine ratio (UACR) and blood urea nitrogen (BUN) levels compared with control mice with saline injection (Table 1). We found that the highest UACR (15 mg/mg) was detected 1 week after BSA injection, followed by a decline (1.6 mg/mg) at 4 weeks and a final rebound (6.3 mg/mg) at 8 weeks. The BUN level was also markedly increased in the BSA group and elevated steadily during the observation period.

Renal cortical expression of AKI markers (KIM-1, NGAL and cystatin C) were upregulated after BSA injection for 1 week. Notably, the expression of KIM-1 and NGAL was further upregulated after 8 weeks of BSA injection (Figure 4b).

### Dynamic Wnt/*β*-catenin signaling in murine protein overload model

**Figure 5:**
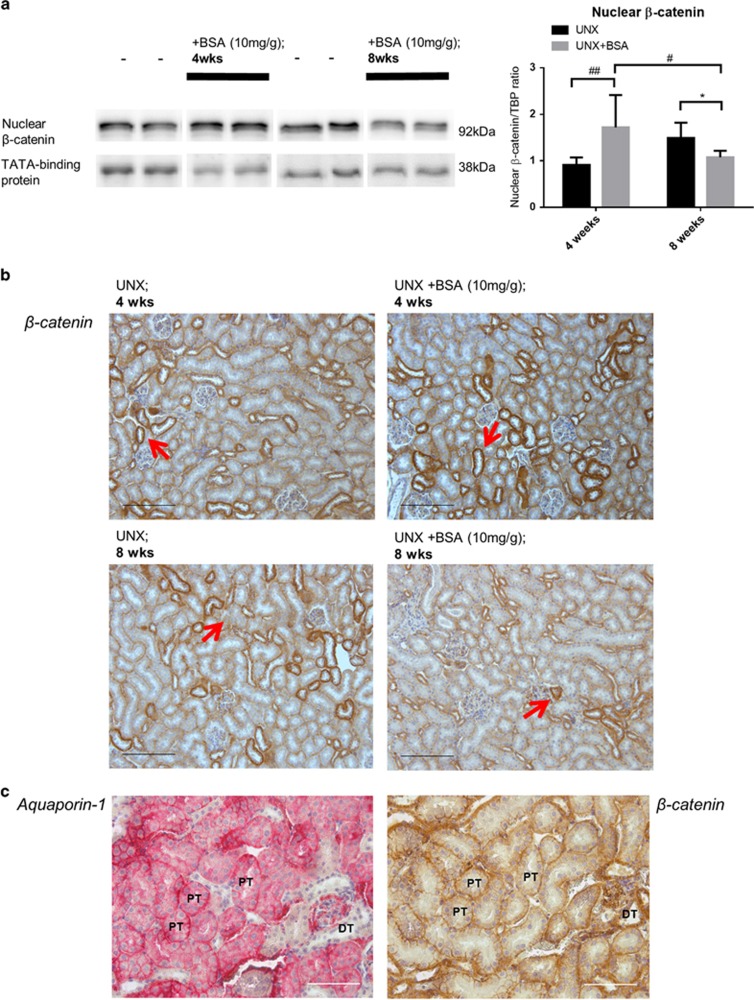
Expression and localization of *β*-catenin in kidney cortex of murine protein overloaded model at different end points. (**a**) Expression of *β*-catenin (*n*=6) in kidney cortical lysates of protein overloaded mice at 4 weeks and 8 weeks. Representative immunoblots are shown. (**b**) Immunohistochemical staining of tubular *β*-catenin (red-arrows) on the respective kidney tissues. Bar scale=200 *μ*m. (**c**) Immunohistochemical stainings of aquaporin-1 (proximal tubule marker) and *β*-catenin on contiguous kidney sections. Bar scale=100 *μ*m. Graphs were expressed in mean± S.D. *P<0.05; ^#^*P*<0.05, ^##^*P*<0.01

The Wnt/*β*-catenin signaling pathway was investigated in the kidney of mice after 4 and 8 weeks of BSA injection. Nuclear *β*-catenin expression in cortical lysates was increased significantly at 4 weeks but decreased at 8 weeks as shown by western blot (Figure 5a). Immunohistochemical staining revealed that *β*-catenin was primarily localized in both renal proximal and distal tubules (Figures 5b and c). These longitudinal and dynamic changes in Wnt/*β*-catenin signaling also correlated with the fluctuations in UACR levels at the corresponding time points (Table 1).

### Renal expression of Dkk-3 was negatively correlated with *β*-catenin expression in murine protein overload model

**Figure 6:**
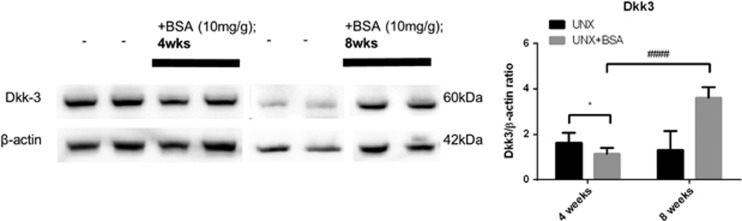
Expression of Dkk-3 in kidney cortical lysate of murine protein overloaded model at different end points. Expression of Dkk-3 (*n*=6) in kidney cortical lysates of protein overloaded mice at 4 weeks and 8 weeks. Graphs were expressed in mean±S.D. *P<0.05; ^####^*P*<0.0001

Renal Dkk-3 expression was downregulated at 4 weeks but upregulated at 8 weeks after BSA injection (Figure 6). The expression level of Dkk-3 was negatively correlated with the level of *β*-catenin at the respective time point of BSA injection. This finding is in line with our *in vitro* results and suggests that upregulation of Dkk-3 is associated with the suppression of *β*-catenin.

### Abrogation of tubular Wnt/*β*-catenin signaling induced tubular cell death in murine protein overload model

**Figure 7:**
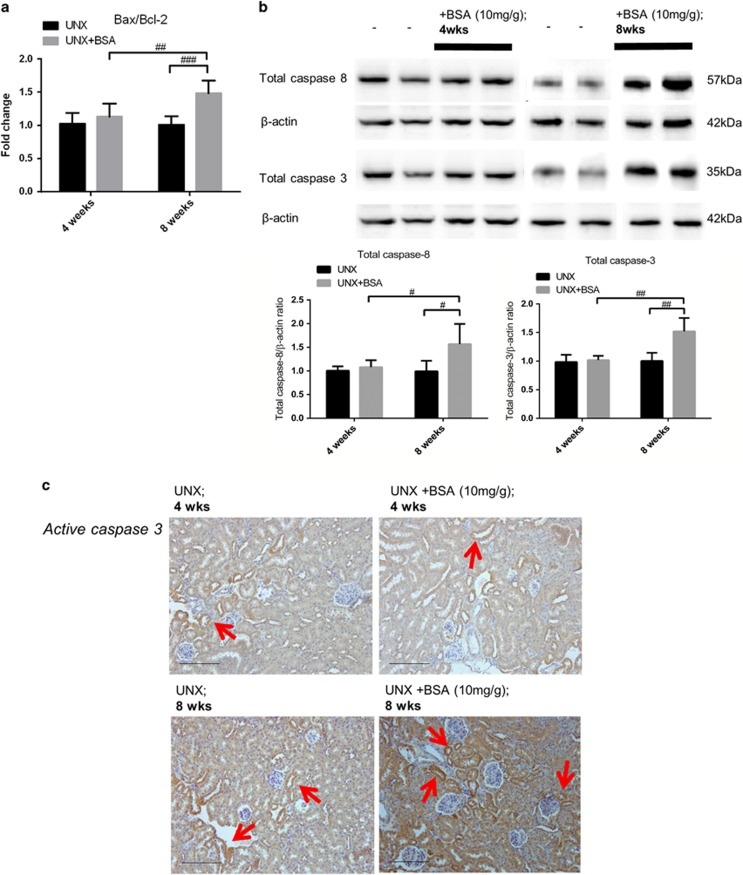

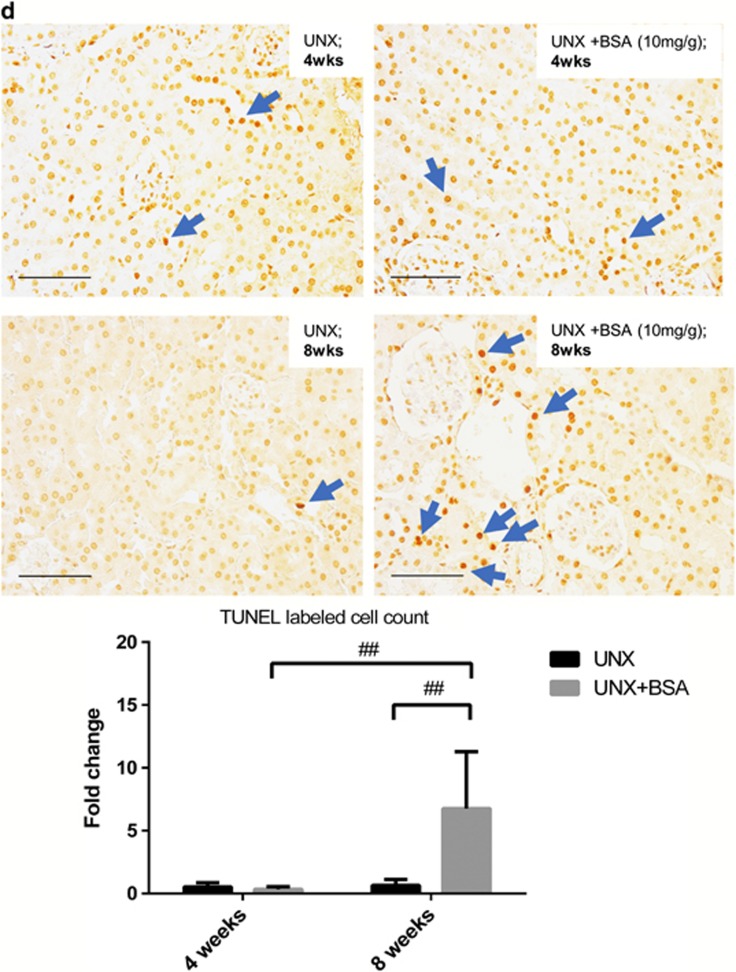
Expression of apoptotic markers in kidney cortex of murine protein overloaded model at different end points. (**a**) Renal cortical Bax/Bcl-2 gene expression ratio measured by quantitative real-time qPCR. (**b**) Expression of total caspase-3 (*n*=6) and 8 (*n*=6) in kidney cortical lysates of protein overloaded mice at 4 weeks and 8 weeks. (**c**) Immunohistochemical staining of active caspase-3 (red arrows) on the respective kidney tissues. Bar scale=200 *μ*m. (**d**) TUNEL-positive cell nuclei (blue arrows) on paraffin kidney sections and fold change of labeled cell counts. Bar scale=100 *μ*m. Graphs were expressed in mean± S.D. ^#^*P*<0.05, ^##^*P*<0.01, ^###^*P*<0.0001

We postulate that downregulated *β*-catenin expression in tubules is detrimental and may exacerbate protein overload damage on tubular injury via induction of cell death. Real-time quantitative PCR showed that the gene expression ratio of Bax/Bcl-2 in the kidney cortex was only upregulated after 8 weeks of BSA injection (Figure 7a). Similarly, the expression of caspase-3 and 8 was also upregulated after 8 weeks of BSA injection (Figure 7b). Apoptosis in renal tubular cells was confirmed by immunohistochemical staining and TUNEL assay at 8 weeks after BSA injection (Figures 7c and d). Collectively, these findings revealed that the increase in tubular cell death coincided with the abrogation of intracellular Wnt/*β*-catenin signaling.

## Discussion

Progressive CKD is effected through a complex multiplicity of tubular cell signalings,^16^ of which canonical Wnt/*β*-catenin signaling pathway has received mounting attention recently. A growing body of research has focused on its pivotal role in a variety of acute and chronic renal pathologies. In particular, the dual roles of Wnt/*β*-catenin signaling – whether it mediates renal injury (destructive) or repair (beneficial) – has been a subject of intense debate.^32^ Here, we reported for the first time the dynamic correlation between protein overload and Wnt/*β*-catenin signaling pathway and suggested that the regulatory protein Dkk-3 may mediate renal tubular apoptosis and the ensuing renal injury.

Besides acting as a transcription factor, *β*-catenin is one of the crucial scaffold proteins that forms a complex with E-cadherin to support the tubular epithelial architecture. It is postulated that *β*-catenin could dissociate from the cell membrane and contribute to the cytosolic-free *β*-catenin equilibrium pool.^33^ Once the cytosolic *β*-catenin is stabilized, it translocates to the cell nucleus and triggers target gene transcription. To mimic the persistent proteinuric state in renal tubules of CKD patients, we used an *in vitro* HK-2 cell culture system with prolonged exposure to HSA treatment (4 days). We first demonstrated that the membrane-bound *β*-catenin expression was reduced in protein overloaded HK-2 cells. The expression of active nuclear *β*-catenin, which drives TCF/LEF promoter activity, was also diminished in PTECs upon the protein stress. These data confirmed that stress from protein overload downregulated Wnt/*β*-catenin signaling pathway in renal tubular cells. In agreement with this, stress from exposure to cyclosporine A or high glucose has also been associated with the suppression of Wnt/*β*-catenin signaling pathway.^34, 35^

Dkk-1 is the most well-known endogenous inhibitor of Wnt/*β*-catenin signaling pathway in different disease models,^36, 37^ however, the role of Dkk-3 is largely unknown. Here, we found that protein overload upregulated Dkk-3 synthesis and secretion in HK-2 cells. Exogenous Dkk-3 protein also inhibited intracellular *β*-catenin expression in an autocrine manner. Our finding that Dkk-3 suppressed tubular Wnt/*β*-catenin signaling in proteinuric nephropathy was in line with other experiment, which showed that Dkk-3 is an inducer of apoptosis^31, 38^ and inhibition of this protein leads to the activation of Wnt/*β*-catenin signaling in lung cancer.^39^ Our findings should ideally be confirmed using cell-specific Dkk-3 knockout animal models, or using a Dkk-3 neutralizing antibody. However, both are currently not readily available.

Wnt/*β*-catenin signaling pathway is known to regulate cell fate, and it is not surprising that its activation allows continuous cell growth as observed in carcinogenesis.^40^ In chronic kidney injury, protein overload causes tubular injury via induction of cell death. Hence, we postulated that downregulation of tubular Wnt/*β*-catenin expression by protein overload promoted tubular apoptosis. Indeed, we found that gene silencing of *β*-catenin in HK-2 cells enhanced HSA-induced apoptosis as demonstrated by the upregulation of apoptotic markers, including the Bax/Bcl-2 gene expression ratio, caspase-3 activity and TUNEL-positive cells.

Wnt/*β*-catenin signaling has been implicated in other disease models that are associated with interstitial inflammation such as diabetes^35^ and the murine model of unilateral ureter obstruction.^37^ In AKI, ablation of *β*-catenin in tubular cells aggravated kidney damage with increasing cell death. Conversely, tubular cells were protected from apoptosis by Wnt1 or stabilization of *β*-catenin^30^ demonstrating the differential roles of *β*-catenin in kidney injury and repair. Here, we showed evidence for the first time that *β*-catenin may have dual roles during longitudinal disease progression in the protein overload model.

In addition to apoptosis, expression levels of AKI markers (KIM-1, NGAL and cystatin C) were also upregulated in the renal cortex of mice with the highest proteinuria at 1 week after protein overload. Interestingly, intrarenal KIM-1 and NGAL expression levels were sustained up to 8 weeks after BSA injection, indicating that the persistent acute injury response was extended to the chronic phase.

Fluctuations in UACR appeared to be associated with the level of *β*-catenin expression. Notably, amelioration of UACR level coincided with the overexpression of tubular *β*-catenin at the early phase (4 weeks). Conversely, elevation of UACR level correlated with the decline in tubular *β*-catenin expression at the late phase (8 weeks). It is noteworthy that the trend of tubular *β*-catenin expression under protein overload was also in line with our *in vitro* results. Together with the observation of increased tubular apoptosis toward the late phase of murine protein overload model, we speculate that downregulation of Wnt/*β*-catenin signaling is a driving force for both proteinuria and tubular cell death. This phenomenon suggests that heightened tubular cell death casts a negative impact on the degree of proteinuria. One possible underlying mechanism of suppressed Wnt/*β*-catenin signaling is the induction of Dkk-3. The expression of Dkk-3 was downregulated at the early stage of the protein overload and upregulated thereafter. In agreement with our *in vitro* data, renal Dkk-3 expression was negatively correlated with tubular *β*-catenin expression at the respective time point, suggesting that prolonged protein overload induced tubular Dkk-3 expression and suppressed tubular *β*-catenin expression.

Taken together, our data support the notion that upregulation of renal Dkk-3 expression in chronic proteinuric nephropathy inhibits tubular Wnt/*β*-catenin signaling pathway that unleashes the downstream detrimental events. Maintenance of tubular *β*-catenin signaling could be renoprotective as it has anti-apoptotic property and partially ameliorates proteinuria. Further investigation is warranted to exploit this pathway in order to unveil new therapeutic targets for proteinuric CKD.

## Materials and Methods

### Cell culture

An immortalized human PTEC cell line derived from normal kidney named HK-2 cells was purchased (ATCC, Manassas, VA, USA). HK-2 cells were grown in Dulbecco's modified Eagle's medium (DMEM/F-12, GlutaMAX; Invitrogen, Carlsbad, CA, USA), supplemented with penicillin (100 IU/ml) and streptomycin (100 *μ*g/ml) and 10% fetal bovine serum (FBS; Invitrogen), at 37 °C and 5% CO_2_. HK-2 cells were grown to subconfluence and rendered in medium without FBS for 24 h prior further treatments. HSA (5 mg/ml) was obtained from CSL laboratory (Parkville, Victoria, Australia) and applied to HK-2 cells for up to 4 days, with culture media changed every 2 days. HK-2 cells were also treated with 0.1 *μ*g/ml human recombinant Dkk-3 (R&D Systems, Minneapolis, MN, USA).

### Mouse model of protein overload nephropathy

This study was approved by the Committee on the Use of Live Animals in Teaching and Research (Laboratory Animal Unit, The University of Hong Kong) and was performed in accordance with the National Institute of Health Guide for the Care and Use of Laboratory Animals. The protocol of protein overload model was carried out according to previous study.^41^ Briefly, 6-week-old C57/BL6 mice weight approximately 20 g were divided into different groups randomly, *ad libitum* to food and water. All mice were uninephrectomized under anesthesia at the age of 6 weeks before intraperitoneal injection of low endotoxin BSA (A-9430, Sigma-Aldrich, St. Louis, MO, USA) at the dose of 10 mg/g body weight for 5 consecutive days weekly, up to 4 and 8 weeks. Control mice were injected with equal volume of saline. Twenty-four-hour urine and blood serum were collected at 1, 4 and 8 weeks after BSA injection. Mice were killed at 4 and 8 weeks after BSA injection and kidney tissues were collected. Each of the kidney tissue was dissected into half; one half was snap-frozen in liquid nitrogen and stored at −70 °C, the other half was fixed with 10% formalin and embedded in paraffin for immunohistochemistry examinations. RNA and protein were extracted from frozen kidney cortices.

### siRNA transfection in HK-2 cells

HK-2 cells were transfected with siRNA targeting CTNNB1 (ID: S438; Applied Biosystems, Carlsbad, CA, USA) by Lipofectamine 2000 (Invitrogen) in Opti-MEM I Reduced Serum medium (Applied Biosystems) for 6 h, followed by incubation with fresh DMEM/F-12, GlutaMAX medium for another 18  h before they were subjected to HSA treatment. The knockdown efficiency was verified by immunoblotting assay.

### RNA isolation and quantitative real-time PCR

Total RNA was isolated from HK-2 cells and mouse kidney cortical tissues using Trizol (Invitrogen) and NucleoSpin Triprep (Macherey-Nagel, Duren, Germany), respectively. RNAs was reversely transcribed according to the manufacturer's instructions (Applied Biosystems). Quantitative real-time PCR was performed using ABI 7500 Real-Time PCR System (Applied Biosystems) with SYBR Green reagent (Applied Biosystems) and specific primers (Table 2). Relative gene expression of individual sample was calculated after normalization with *β*-actin expression. All experimental groups were compared with their respective control group using SDS software (Applied Biosystems).

### Immunoblotting assay

Total protein, cytosolic and nuclear protein were extracted from HK-2 cells using lysis buffer and NE-PER extraction kit (Thermo Scientific, Rockford, IL, USA) respectively, containing protease inhibitor cocktail (Sigma-Aldrich). For mouse kidney tissue, total protein were extracted by NucleoSpin Triprep or fractionized by the NE-PER extraction kit. Protein concentration was quantified by BCA Protein Assay Kit (Thermo Scientific). Equal amount of protein was resolved by 12% SDS-PAGE and then transferred to PVDF membranes. After blocking with 5% non-fat milk, the membranes were subjected to overnight primary antibody incubation using antibody against active *β*-catenin, total caspase-3, total caspase-8 (Cell Signalling Technology, Beverly, CA, USA), Dicckopf-3, TATA-binding protein (TBP; Abcam, Cambridge, UK) and *β*-actin (Thermo Scientific). The membranes were then incubated with peroxidase conjugated secondary antibodies (Dako, Carpinteria, CA, USA) accordingly and visualized with ECL prime chemiluminescnece (GE Healthcare, Buckinghamshire, UK) by using ChemiDoc XRS+ system (Bio-Rad, Hercules, CA, USA). Protein expression levels were quantified and normalized to *β*-actin/TBP by Image Lab software (Bio-Rad).

### ELISA

The level of Dkk-3 from HK-2 culture medium was measured by a commercial ELISA kit (Abcam). Mouse urinary albumin was measured by mouse albumin ELISA quantitation kit (Bethyl Laboratories, Montgomery, TX, USA). Urine creatinine and serum BUN were quantified by enzymatic assays (Stanbio Laboratory, Boerne, TX, USA).

### Caspase-3 activity assay

HK-2 cells were subjected to the albumin or siRNA treatment as described and lysed for caspase-3 activity assay, according to the manufacturer's protocol of ApoAlert Caspase Colorimetric Assay Kit (Clontech Laboratories, Mountain View, CA, USA). Briefly, the cell lysates were incubated with the chromophore p-nitroaniline (pNA) linked with the specific caspase-3 substrate. Caspase-3 activity was measured by spectrophotometric detection of released pNA after the cleavage by active caspase-3.

### Immunohistochemical staining

Paraffin-embedded kidney tissue sections (4 *μ*m) were de-paraffinized, rehydrated with concentration gradient of ethanol. The sections were subjected to antigen retrieval step, by microwave-based method in 10 mM citrate buffer, or incubation with 20 ug/ml proteinase k (Qiagen, Valencia, CA, USA) for 10 min Then, the sections were quenched in 3% hydrogen peroxide for 10 min and incubated with 2% BSA to block nonspecific binding. Primary antibodies against *β*-catenin (BD Bioscience, San Jose, CA, USA), aquaporin-1 and active caspase-3 (Abcam) were added to the sections overnight and subsequently with secondary peroxidase conjugated antibodies (Dako) or alkaline phosphatase conjugated antibodies (Enzo Life Sciences, Farmingdale, NY, USA) for 1 h. The sections were developed by using DAB substrate from the Envision Plus system (Dako) or HIGHDEF red chromogen (Enzo Life Sciences), followed by counterstain with CAT hematoxylin (Biocare Medical, Walnut Creek, CA, USA). The slides were then dehydrated with ethanol and xylene before mounting.

### Immunofluorescence staining

HK-2 cells were seeded on chamber slides (Thermo Scientific) and incubated with HSA or Dkk-3 treatments. Cells were fixed with 4% paraformaldehyde (Sigma-Aldrich), followed by permeabilization in 0.25% Triton X 100 and 2% BSA (Sigma-Aldrich) incubation. The slides were then incubated in primary antibody against *β*-catenin (BD Bioscience) overnight thereafter incubated with the corresponding FITC-conjugated secondary antibody (Dako). The chamber slides were mounted with ProLong antifade mounting medium (Invitrogen) and examined under fluorescence microscope (400X; Leica, Bensheim, Germany).

### Luciferase reporter assay

Nano-Glo Dual-Luciferase Reporter Assay System (Promega, Madison, WI, USA) was used to measure the TCF/LEF promoter activities. HK-2 cells were seeded in 96-well plate and co-transfected with pGL4.53 firefly luciferase (Fluc) and pGL4 (luc2CP/TCF-LEF RE/Hygro) NanoLuc luciferase (Nluc) plasmid using Lipofectamine 2000 (Invitrogen). Transfected cells were treated HSA and Dkk-3 for the respective duration lengths, then lysed with lysis buffer with Fluc substrate and incubated on an orbital shaker for 10 min. Fluc signals were then quenched and following by reaction with Nluc substrate. The signals in arbitrary unit (AU) from both Nluc and Fluc were measured by a luminometer (BMG Labtech GmbH, Ortenberg, Germany). The promoter activities were calculated by the ratio of respective AU values of Nluc/Fluc.

### Albumin uptake

FITC-conjugated HSA, 1 mg/ml (Jackson ImmunoResearch Laboratories, Inc., West Grove, PA, USA) was treated to HK-2 cells for albumin uptake measurement. HK-2 cells were seeded in 96-well black plate with clear bottom and treated with the labeled albumin for the respective time points. Cells were lysed with 0.1% SDS and the fluorescence intensities were measured by a FLUOstar Omega Microplate Reader (BMG Labtech).

### TUNEL assay

#### *In vitro* assay

HK-2 cells were seeded in chamber slides and treated with HSA or siRNA transfection. Apoptotic cells were detected by ApopTag Peroxidase *In Situ* Apoptosis Detection Kit (Millipore, Bedford, MA, USA). The cells were fixed with 4% paraformaldehyde (Sigma-Aldrich), permeabilized with ethanol:acetic acid, 2 : 1 (v : v) and followed by protocol according to the manufacturer's instructions. Cells were counterstained with Methyl Green (Dako) and were examined under light microscopy (200X). Images were taken on non-overlap areas, TUNEL-labeled cells/total cell number ratios were counted for individual image and averaged for each experimental sample.

#### *In vivo* assay

Deparaffinized, rehydrated and antigen retrieved sections of mouse tissue were subjected to apoptotic analysis by *In Situ* Cell Death Detection kit, POD (Roche Applied Sciences, Indianapolis, IN, USA). For mouse tissue sections, approximately 10 images of cortical area with at least one glomerulus (400X) were taken. TUNEL-labeled cells/total cell number ratios were counted for individual image and averaged for each experimental sample.

### Statistical analysis

All data were expressed as mean± S.D. from three independent experiments. Two/one-way ANOVA with Tukey's multiple comparison test or *t*-test were used to calculate the difference between experimental groups. GraphPad Prism v.4 (GraphPad Software, San Diego, CA, USA) was used to evaluate the data. *P*<0.05 was considered statistically significant.

## Figures and Tables

**Table 1:** Urine and serum biochemistry data of UNX C57/BL6 murine receiving 10 mg/g BSA for 4 or 8 weeks

**BSA injection period**	**1 Week**		**4 Weeks**		**8 Weeks**	
	**UNX**	**UNX+ BSA (10 mg/g)**	**UNX**	**UNX+ BSA (10 mg/g)**	**UNX**	**UNX+ BSA (10 mg/g)**
UACR (*μ*g/mg)	270.8±88.8^a^	15 677.3±5009.6^a^	102.7±16.2	1621.8±768.4^a,b^	112.9±8.7^c^	6341.2±1486.6^b,c^
BUN (mg/dl)	29.8±2.7	35.0±7.3^c^	29.3±1.6^a^	46.0±7.2^a,c^	29.3±1.9^a^	49.1±1.9^a,c^

Abbreviations: BSA, bovine serum albumin; BUN, blood urea nitrogen; UACR, urine albumin-to-creatinine ratio; UNX, uninephrectomized

UACR and BUN were measured for the experimental animals (*n*=6) and expressed in mean±S.D. a=*P*<0.0001; b=*P*<0.05; c=*P*<0.001

**Table 2:** Primers designed for quantitative real-time PCR

**Genes**	**Primers**	
	**Forward**	**Reverse**
*Human* DKK-3	5′-GTAAGTTTCCCCTCTGGCTTG-3′	5′-AAGCACCAGACTGTGAAGCCT-3′
*Human* BAX	5′-TTCTGACGGCAACTTCAACTGG-3′	5′-AGGAAGTCCAATGTCCAGCC-3′
*Human* BCL-2	5′-GATGGGAACACTGGTGGAGGATGG-3′	5′-TCTGGAGGGCCCACGGCAG-3′
*Human β*-actin	5′-TGACGTGGACATCCGCAAAG-3′	5′-CTGGAAGGTGGACAGCGAGG-3′
*Mouse* NGAL	5′-ACAACCAGTTCGCCATGGTA-3′	5′-AAGCGGGTGAAACGTTCCTT-3′
*Mouse* KIM-1	5′-GTCGTGGGTCTTCCTGTACTC-3′	5′-AAACCAGAGATTCCCACACG-3′
*Mouse* cystatin C	5′-TGTTTGCACCAGGAGACAGT-3′	5′-AATTGAGCAAGGCATGGCAG-3′
*Mouse* Bax	5′-CCCGAGCTGATCAGAACCAT-3′	5′-GGGGTCCCGAAGTAGGAGAG-3′
*Mouse* Bcl-2	5′-CTTTGAGTTCGGTGGGGTCA-3′	5′-AGTTCCACAAAGGCATCCCA-3′
*Mouse β*-actin	5′-TCCATCATGAAGTGTGACGT-3′	5′-GAGCAATGATCTTGATCTTCAT-3′

## Abbreviations

AKI: acute kidney injury
BUN: blood urea nitrogen
BSA: bovine serum albumin
CKD: chronic kidney disease
DN: diabetic nephropathy
Dkk-3: Dickkopf-3
EMT: epithelial–mesenchymal transition
HK-2 cells: human kidney-2 cells
HSA: human serum albumin
Lrp: LDL receptor-related protein
PTECs: proximal tubular epithelial cells
TCF/LEF: T-cell factor and lymphoid enhancer-binding factor
TUNEL: terminal deoxynucleotidyl transferase dUTP nick-end labeling
UACR: urine albumin-to-creatinine ratio

## References

[bib1] Hallan SI, Ritz E, Lydersen S, Romundstad S, Kvenild K, Orth SR. Combining GFR and albuminuria to classify CKD improves prediction of ESRD. J Am Soc Nephrol 2009; 20: 1069–1077. 19357254 10.1681/ASN.2008070730 PMC2678033

[bib2] Ruggenenti P, Cravedi P, Remuzzi G. Mechanisms and treatment of CKD. J Am Soc Nephrol 2012; 23: 1917–1928. 23100218 10.1681/ASN.2012040390

[bib3] Christensen EI, Birn H. Megalin and cubilin: multifunctional endocytic receptors. Nat Rev Mol Cell Biol 2002; 3: 256–266. 11994745 10.1038/nrm778

[bib4] Birn H, Christensen EI. Renal albumin absorption in physiology and pathology. Kidney Int 2006; 69: 440–449. 16514429 10.1038/sj.ki.5000141

[bib5] Zoja C, Benigni A, Remuzzi G. Cellular responses to protein overload: key event in renal disease progression. Curr Opin Nephrol Hypertens 2004; 13: 31–37. 15090857 10.1097/00041552-200401000-00005

[bib6] Abbate M, Zoja C, Remuzzi G. How does proteinuria cause progressive renal damage? J Am Soc Nephrol 2006; 17: 2974–2984. 17035611 10.1681/ASN.2006040377

[bib7] Imai E, Nakajima H, Kaimori JY. Albumin turns on a vicious spiral of oxidative stress in renal proximal tubules. Kidney Int 2004; 66: 2085–2087. 15496183 10.1111/j.1523-1755.2004.00044.x

[bib8] Tang S, Leung JC, Abe K, Chan KW, Chan LY, Chan TM et al. Albumin stimulates interleukin-8 expression in proximal tubular epithelial cells *in vitro* and *in vivo*. J Clin Invest 2003; 111: 515–527. 12588890 10.1172/JCI16079 PMC151921

[bib9] Wu HJ, Yiu WH, Li RX, Wong DW, Leung JC, Chan LY et al. Mesenchymal stem cells modulate albumin-induced renal tubular inflammation and fibrosis. PLoS One 2014; 9: e90883. 24646687 10.1371/journal.pone.0090883 PMC3960109

[bib10] Li X, Pabla N, Wei Q, Dong G, Messing RO, Wang CY et al. PKC-delta promotes renal tubular cell apoptosis associated with proteinuria. J Am Soc Nephrol 2010; 21: 1115–1124. 20395372 10.1681/ASN.2009070760 PMC3152238

[bib11] Caruso-Neves C, Pinheiro AA, Cai H, Souza-Menezes J, Guggino WB. PKB and megalin determine the survival or death of renal proximal tubule cells. Proc Natl Acad Sci USA 2006; 103: 18810–18815. 17121993 10.1073/pnas.0605029103 PMC1693744

[bib12] Wu X, He Y, Jing Y, Li K, Zhang J. Albumin overload induces apoptosis in renal tubular epithelial cells through a CHOP-dependent pathway. OMICS 2010; 14: 61–73. 20141329 10.1089/omi.2009.0073

[bib13] Sanz AB, Santamaria B, Ruiz-Ortega M, Egido J, Ortiz A. Mechanisms of renal apoptosis in health and disease. J Am Soc Nephrol 2008; 19: 1634–1642. 18632846 10.1681/ASN.2007121336

[bib14] Linkermann A, Chen G, Dong G, Kunzendorf U, Krautwald S, Dong Z. Regulated cell death in AKI. J Am Soc Nephrol 2014; 25: 2689–2701. 24925726 10.1681/ASN.2014030262 PMC4243360

[bib15] Erkan E, De Leon M, Devarajan P. Albumin overload induces apoptosis in LLC-PK(1) cells. Am J Physiol Renal Physiol 2001; 280: F1107–F1114. 11352849 10.1152/ajprenal.2001.280.6.F1107

[bib16] Baines RJ, Brunskill NJ. Tubular toxicity of proteinuria. Nat Rev Nephrol 2011; 7: 177–180. 21151210 10.1038/nrneph.2010.174

[bib17] MacDonald BT, Tamai K, He X. Wnt/beta-catenin signaling: components, mechanisms, and diseases. Dev Cell 2009; 17: 9–26. 19619488 10.1016/j.devcel.2009.06.016 PMC2861485

[bib18] Schmidt-Ott KM, Barasch J. WNT/beta-catenin signaling in nephron progenitors and their epithelial progeny. Kidney Int 2008; 74: 1004–1008. 18633347 10.1038/ki.2008.322 PMC2909845

[bib19] Moon RT, Kohn AD, De Ferrari GV, Kaykas A. WNT and beta-catenin signalling: diseases and therapies. Nat Rev Genet 2004; 5: 691–701. 15372092 10.1038/nrg1427

[bib20] Clevers H, Nusse R. Wnt/beta-catenin signaling and disease. Cell 2012; 149: 1192–1205. 22682243 10.1016/j.cell.2012.05.012

[bib21] Rao TP, Kuhl M. An updated overview on Wnt signaling pathways: a prelude for more. Circ Res 2010; 106: 1798–1806. 20576942 10.1161/CIRCRESAHA.110.219840

[bib22] Nusse R, Varmus H. Three decades of Wnts: a personal perspective on how a scientific field developed. EMBO J 2012; 31: 2670–2684. 22617420 10.1038/emboj.2012.146 PMC3380217

[bib23] Kawano Y, Kypta R. Secreted antagonists of the Wnt signalling pathway. J Cell Sci 2003; 116(Pt 13): 2627–2634. 12775774 10.1242/jcs.00623

[bib24] Lancaster MA, Louie CM, Silhavy JL, Sintasath L, Decambre M, Nigam SK et al. Impaired Wnt-beta-catenin signaling disrupts adult renal homeostasis and leads to cystic kidney ciliopathy. Nat Med 2009; 15: 1046–1054. 19718039 10.1038/nm.2010 PMC2895985

[bib25] Wang W, Li F, Sun Y, Lei L, Zhou H, Lei T et al. Aquaporin-1 retards renal cyst development in polycystic kidney disease by inhibition of Wnt signaling. FASEB J 2015; 29: 1551–1563. 25573755 10.1096/fj.14-260828 PMC4396615

[bib26] Uhlenhaut NH, Treier M. Transcriptional regulators in kidney disease: gatekeepers of renal homeostasis. Trends Genet 2008; 24: 361–371. 18514358 10.1016/j.tig.2008.05.001

[bib27] Liu YH. New insights into epithelial-mesenchymal transition in kidney fibrosis. J Am Soc Nephrol 2010; 21: 212–222. 20019167 10.1681/ASN.2008121226 PMC4554339

[bib28] Maarouf OH, Ikeda Y, Humphreys BD. Wnt signaling in kidney tubulointerstitium during disease. Histol Histopathol 2015; 30: 163–171. 25297005 10.14670/HH-30.163

[bib29] Nelson PJ, von Toerne C, Grone HJ. Wnt-signaling pathways in progressive renal fibrosis. Expert Opin Ther Targets 2011; 15: 1073–1083. 21623684 10.1517/14728222.2011.588210

[bib30] Zhou D, Li Y, Lin L, Zhou L, Igarashi P, Liu Y. Tubule-specific ablation of endogenous beta-catenin aggravates acute kidney injury in mice. Kidney Int 2012; 82: 537–547. 22622501 10.1038/ki.2012.173 PMC3425732

[bib31] Mizobuchi Y, Matsuzaki K, Kuwayama K, Kitazato K, Mure H, Kageji T et al. REIC/Dkk-3 induces cell death in human malignant glioma. Neuro Oncol 2008; 10: 244–253. 18443132 10.1215/15228517-2008-016 PMC2563047

[bib32] Kawakami T, Ren S, Duffield JS. Wnt signalling in kidney diseases: dual roles in renal injury and repair. J Pathol 2013; 229: 221–231. 23097132 10.1002/path.4121

[bib33] Howard S, Deroo T, Fujita Y, Itasaki N. A positive role of cadherin in Wnt/beta-catenin signalling during epithelial-mesenchymal transition. PLoS One 2011; 6: e23899. 21909376 10.1371/journal.pone.0023899 PMC3166074

[bib34] Berzal S, Alique M, Ruiz-Ortega M, Egido J, Ortiz A, Ramos AM. GSK3, snail, and adhesion molecule regulation by cyclosporine A in renal tubular cells. Toxicol Sci 2012; 127: 425–437. 22416070 10.1093/toxsci/kfs108

[bib35] Ho C, Lee PH, Hsu YC, Wang FS, Huang YT, Lin CL. Sustained Wnt/beta-catenin signaling rescues high glucose induction of transforming growth factor-beta1-mediated renal fibrosis. Am J Med Sci 2012; 344: 374–382. 22270399 10.1097/MAJ.0b013e31824369c5

[bib36] He W, Tan RJ, Li Y, Wang D, Nie J, Hou FF et al. Matrix metalloproteinase-7 as a surrogate marker predicts renal Wnt/beta-catenin activity in CKD. J Am Soc Nephrol 2012; 23: 294–304. 22095947 10.1681/ASN.2011050490 PMC3269179

[bib37] He W, Dai C, Li Y, Zeng G, Monga SP, Liu Y. Wnt/beta-catenin signaling promotes renal interstitial fibrosis. J Am Soc Nephrol 2009; 20: 765–776. 19297557 10.1681/ASN.2008060566 PMC2663839

[bib38] Xiang T, Li L, Yin X, Zhong L, Peng W, Qiu Z et al. Epigenetic silencing of the WNT antagonist Dickkopf 3 disrupts normal Wnt/beta-catenin signalling and apoptosis regulation in breast cancer cells. J Cell Mol Med 2013; 17: 1236–1246. 23890219 10.1111/jcmm.12099 PMC4159020

[bib39] Yue W, Sun Q, Dacic S, Landreneau RJ, Siegfried JM, Yu J et al. Downregulation of Dkk3 activates beta-catenin/TCF-4 signaling in lung cancer. Carcinogenesis 2008; 29: 84–92. 18048388 10.1093/carcin/bgm267

[bib40] Kruck S, Eyrich C, Scharpf M, Sievert KD, Fend F, Stenzl A et al. Impact of an altered Wnt1/beta-catenin expression on clinicopathology and prognosis in clear cell renal cell carcinoma. Int J Mol Sci 2013; 14: 10944–10957. 23708097 10.3390/ijms140610944 PMC3709711

[bib41] Eddy AA, Kim H, Lopez-Guisa J, Oda T, Soloway PD. Interstitial fibrosis in mice with overload proteinuria: deficiency of TIMP-1 is not protective. Kidney Int 2000; 58: 618–628. 10916085 10.1046/j.1523-1755.2000.00208.x

